# Study on the Preparation and PEC-Type Photodetection Performance of β-Bi_2_O_3_ Thin Films

**DOI:** 10.3390/ma17153779

**Published:** 2024-08-01

**Authors:** Jiaji Zhang, Zhihua Xiong, Zi Wang, Jinlong Sun

**Affiliations:** 1 Sanya Science and Education Innovation Park, Wuhan University of Technology, Sanya 572025, China; zhangjiaji@whut.edu.cn (J.Z.); 335255@whut.edu.cn (Z.X.); 2 School of Civil Engineering and Architecture, Wuhan University of Technology, Wuhan 430070, China; 3 Hainan Yourui Cohesion Technology Co., Ltd., Sanya 572025, China; 4 Birmingham Centre for Energy Storage & School of Chemical Engineering, University of Birmingham, Birmingham B152TT, UK; 5 School of Art and Design, Wuhan University of Technology, Wuhan 430070, China; zitawang@whut.edu.cn; 6 School of Telecommunications and Information Engineering, Nanjing University of Posts and Telecommunications, Nanjing 210003, China

**Keywords:** β-Bi_2_O_3_ thin films, vapor deposition method, photoelectric response, preparation process

## Abstract

Bismuth-based compounds have been regarded as a kind of promising material due to their narrow bandgap, high carrier mobility, low toxicity, and strong oxidation ability, showing potential applications in the field of photoelectrochemical (PEC) activities. They can be applied in sustainable energy production, seawater desalination and treatment, optical detection and communication, and other fields. As a member of the broader family of bismuth-based materials, β-Bi_2_O_3_ exhibits significant advantages for applications in engineering, including high photoelectric response, stability in harsh environments, and excellent corrosion resistance. This paper presents the synthesis of β-Bi_2_O_3_ thin films utilizing the mist chemical vapor deposition (CVD) method at the optimal temperature of 400 °C. Based on the β-Bi_2_O_3_ thin film synthesized at optimal temperature, a PEC-type photodetector was constructed with the highest responsivity R of 2.84 mA/W and detectivity D of 6.01 × 10^10^ Jones, respectively. The photodetection performance was investigated from various points like illumination light wavelength, power density, and long-term stability. This study would broaden the horizontal and practical applications of β-Bi_2_O_3_.

## 1. Introduction

Bismuth-based materials, due to their unique physical and chemical properties, have shown extensive application prospects in various fields [1,2]. These materials are garnering significant attention from researchers and industry professionals alike because of their versatility and efficiency. Particularly as catalysis, electrical, optical, and thermoelectric materials, bismuth-based materials exhibit superior performance [3,4]. Among the many bismuth-based materials, bismuth trioxide (Bi_2_O_3_) has become one of the research hotspots due to its unique crystal structure and excellent optical and electrical properties [5,6]. This compound’s distinctive characteristics make it ideal for various high-tech and engineering applications, including photovoltaics, sensors, and advanced electronic devices. As research continues, the full potential of Bi_2_O_3_ is expected to be unveiled, contributing significantly to scientific and technological progress [7].

Bi_2_O_3_ exists in various crystalline phases, with the β phase (β-Bi_2_O_3_) being a significant metastable form [8]. This phase features a wide bandgap of approximately 2.8 eV, a high light absorption coefficient (>10^−5^ cm^−1^ for the visible light), exceptional photoelectric conversion efficiency (higher than 10%), a high dielectric constant, and good conductivity. These properties result in high responsivity and quick response times in photodetectors. Meanwhile, its high conductivity enables it to quickly transmit photogenerated carriers in photodetectors, reducing energy loss and improving the signal-to-noise ratio [9]. The β-Bi_2_O_3_ phase’s narrow bandgap (around 2.3 eV) and optimal conduction and valence band positions make it a promising candidate for photocatalysts used in water splitting and various oxidation–reduction reactions [5]. Additionally, β-Bi_2_O_3_ has a tunnel-like structure that facilitates efficient photogenerated carrier transfer due to its anisotropic nature [10]. M. Kim et al. demonstrated that textured β-Bi_2_O_3_, with its short charge transfer pathways, exhibited high stability and a photocurrent density of 0.97 mA cm^−2^ at 0.5 V vs. Ag/AgCl [11].

Currently, the preparation methods for β-Bi_2_O_3_ thin films include solid-state reaction, solution methods, chemical vapor deposition (CVD), and physical vapor deposition (PVD). In general, β-Bi_2_O_3_ nanocrystals were prepared using bismuth nitrate and bismuth oxalate through various solution-based methods [12,13]. The production of β-Bi_2_O_3_ encounters several challenges due to its distinct crystal structure and properties. As a metastable phase, β-Bi_2_O_3_ tends to convert into the more thermodynamically stable α-Bi_2_O_3_ at elevated temperatures. Maintaining pure β-Bi_2_O_3_ without phase transformation can be difficult and often necessitates precise synthesis conditions and stabilizing additives to preserve the β-phase [5]. The summary of the reported preparation methods for β-Bi_2_O_3_ thin films is shown in Table 1.

Due to their high sensitivity and low noise performance, photoelectrochemical (PEC) photodetectors exhibit significant advantages in the field of photodetection [17]. Additionally, the ability of PEC photodetectors to operate over a wide spectral range, coupled with their simple structure and low manufacturing costs, endows them with high practicality and extensive applications in engineering fields [18].

In recent years, research on PEC photodetectors has focused on material selection and structural design. The development and introduction of new materials, in particular, have greatly enhanced the performance of PEC photodetectors. Among these materials, β-Bi_2_O_3_ (beta-bismuth oxide) has emerged as a highly promising candidate due to its unique physicochemical properties. β-Bi_2_O_3_ possesses a wide band gap (approximately 2.85 eV), which allows it to exhibit excellent light absorption capabilities in both the ultraviolet and visible light ranges. Furthermore, β-Bi_2_O_3_ demonstrates high photoelectric conversion efficiency with an IPCE higher than 10% and stability, which are crucial for improving the sensitivity and reliability of photodetectors [19].

Another significant advantage of β-Bi_2_O_3_ lies in its superior electron transport properties, which help reduce the recombination of photogenerated electron–hole pairs, thereby enhancing the generation efficiency of photocurrents [20]. Additionally, the synthesis of β-Bi_2_O_3_ is relatively simple and cost-effective, with various preparation methods available, such as solution methods, thermal evaporation, and CVD [21,22].

Therefore, PEC photodetectors exhibit significant advantages in terms of sensitivity, operational range, and cost [23]. In particular, β-Bi_2_O_3_, with its outstanding photoelectric properties and ease of preparation, has become a material of great interest in this field and is expected to play an increasingly important role in future research and applications.

This paper synthesized β-Bi_2_O_3_ thin films using the mist CVD method. By controlling deposition parameters and annealing temperature, this research aimed to optimize the preparation process of β-Bi_2_O_3_ thin films and improve the crystallization quality and surface morphology of the films. As far as we know, mist CVD is one of the most economical film deposition techniques, and this is the first report of β-Bi_2_O_3_ thin film PEC-type photodetectors, which would attract the attention of industrial staff to follow it, thereby enhancing their photoelectric response performance, and making their performance in practical applications more outstanding.

## 2. Experimental Details

### 2.1. Thin Film Deposition

**Figure 1:**
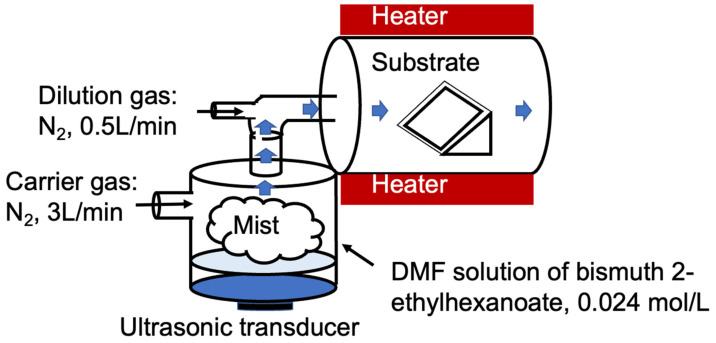
Schematical image of mist CVD for β-Bi_2_O_3_ thin films.

This study synthesized β-Bi_2_O_3_ thin films using mist CVD. The main chemical raw materials and equipment are shown in Table 2 and Figure 1.

In this study, β-Bi_2_O_3_ thin films were prepared on ITO (thickness 200 nm, resistance ≤ 6 Ω)-conductive glass substrates using the mist CVD method. To avoid the influence of water and oxygen in the air, the precursor materials were weighed in an inert environment glove box, and a 0.1 mol/L 25 wt% bismuth(III) 2-ethylhexanoate solution was prepared using DMF as the Bi source. The ITO glass was ultrasonically cleaned sequentially with acetone and ethanol for 15 min each in an ultrasonic cleaner to remove organic impurities from the substrate surface. After cleaning, the substrate was dried with nitrogen (N_2_) and set aside. The cleaned and dried ITO glass was then placed into a tube furnace and heated to a deposition temperature of 400 °C. Once the temperature of the tube furnace stabilized, nitrogen gas (N_2_) was introduced at a flow rate of 1 L/min for 15 min to provide an inert environment. Next, the precursor solution was poured into a nebulizing chamber and dispersed into droplets of approximately 5 µm using an ultrasonic device with a frequency of 2.4 MHz. Finally, driven by N_2_ at a flow rate of 3 L/min, it entered the tube furnace and was diluted with 0.5 L/min of N_2_. The reaction was maintained at a constant temperature for 15 min to obtain the β-Bi_2_O_3_ sample. After the reaction, the sample was cooled to room temperature under a nitrogen atmosphere before being taken out.

### 2.2. Characterization

The crystal structure and phase of the thin films were analyzed using X-ray diffraction (XRD) and Raman spectroscopy. The XRD instrument used was a Bruker D8 Discover (Bremen, Germany), with a copper target and a scanning angle range of 20–60°. The Raman spectrometer used was a Hitachi LamRam HR Evolution (Tokyo, Japan), with a laser wavelength of 532 nm and a test wavenumber range of 50–200 cm^−1^. Using an optical microscope (OM) to analyze the macroscopic surface morphology, the model is C3230BE (CG300) from Shanghai Precision Instrument Co., Ltd. (Shanghai, China). The transmittance and reflectance were measured using a UV-Vis-NIR spectrophotometer, specifically the Lambda 750 S from PerkinElmer. All electrochemical tests were conducted using a CHI660E electrochemical workstation from Chenhua Co., Ltd. (Shanghai, China). The prepared film, platinum electrode, and Ag/AgCl electrode served as the working electrode, counter electrode, and reference electrode, respectively. The effective test area of the working electrode was 1 cm^2^, with a distance of approximately 1 cm between the working and counter electrodes. KOH solution (0.5 mol L^−1^) saturated by N_2_ gas was used as the electrolyte according to the test requirements. LED lights with different wavelengths (365 nm, 455 nm, 520 nm, and 630 nm) and various power levels (ranging from 3 to 90 mW/cm^2^) were used as excitation light sources. The instantaneous current–time (i-t) curves were measured under a bias voltage ranging from 0 V to 0.6 V vs. RHE.

## 3. Results and Discussions

### 3.1. Phase Structure Analysis

**Figure 2:**
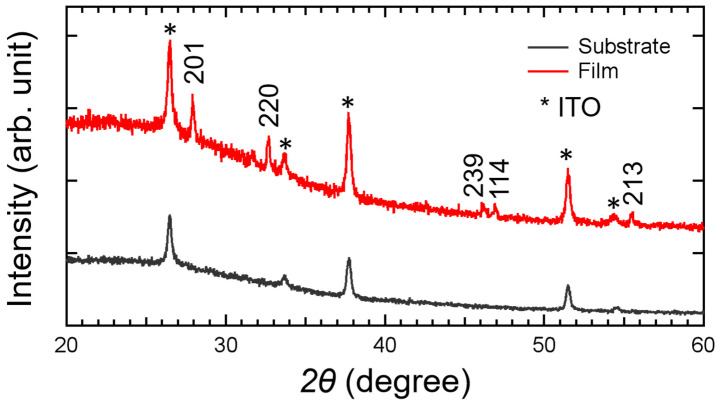
XRD pattern of β-Bi_2_O_3_ thin film synthesized at 400 °C.

To characterize the phase structure of the prepared samples, XRD testing was conducted on β-Bi_2_O_3_, and the results are shown in Figure 2. After comparing with the standard PDF card for β-Bi_2_O_3_ (01-077-5341), it can be observed that, aside from the diffraction peaks from the ITO glass substrate, the β-Bi_2_O_3_ sample shows diffraction peaks at 27.9°, 33.7°, 46.1°, 46.9°, and 55.5°, corresponding to the (201), (220), (239), (114), and (213) crystal planes of β-Bi_2_O_3_, respectively. No diffraction peaks of other substances were found, which fully indicates that the synthesized film is β-Bi_2_O_3_ with high purity. In addition, we calculated the lattice constant and grain size based on the Scherrer formula and Bragg equation, which was summarized in Table 3. The lattice constant is close to the theoretical values.

**Figure 3:**
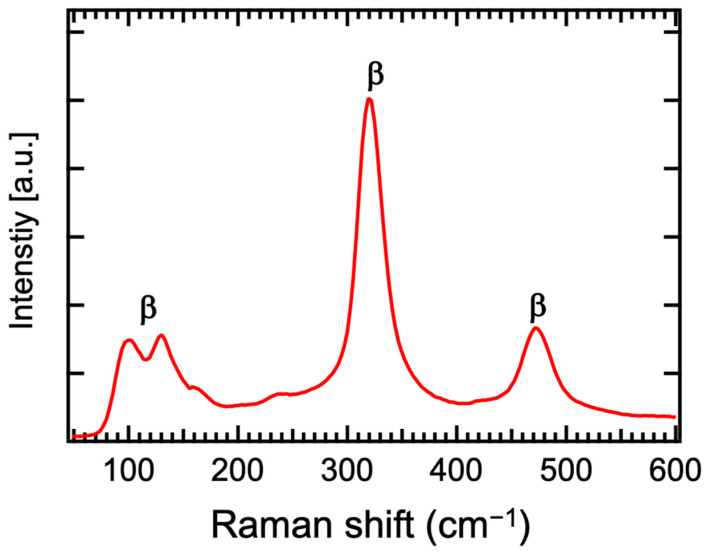
Raman spectra of β-Bi_2_O_3_ thin films synthesized at 400 °C.

Usually, the β phase (201) peak (27.93°), α phase (012) peak (28.03°) (PDF: 01-070-8243), and δ phase (111) peak (27.95°) (PDF: 00-027-0052) have similar two values, so that it is not accurate to determine the crystal structure only based on the XRD θ-2θ patterns. In order to further confirm the crystal structure, the Raman spectra are necessary as these three phases have totally different Raman peaks. Figure 3 reveals from the Raman spectra of the β-Bi_2_O_3_ that all the peaks located at 119, 311 and 463 cm^−1^ in the pattern of the sample grown at 400 °C were well assigned to the β-Bi_2_O_3_ [24,25,26].

### 3.2. Surface Morphology

**Figure 4:**
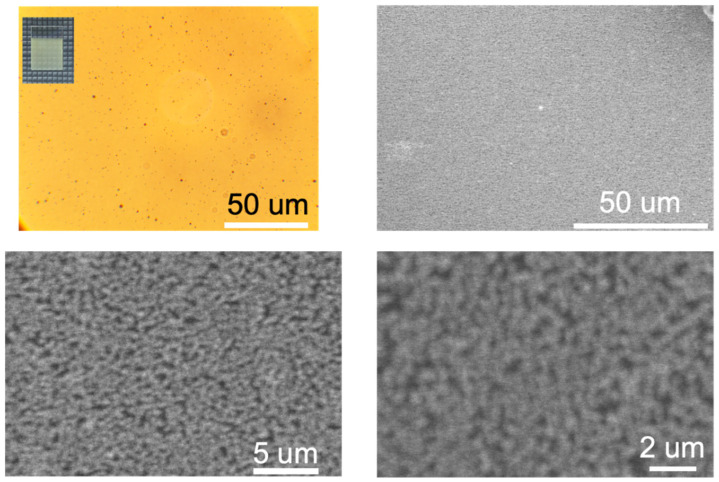
Optical microscope and SEM image with various scale of β-Bi_2_O_3_ thin film synthesized at 400 °C.

**Figure 5:**
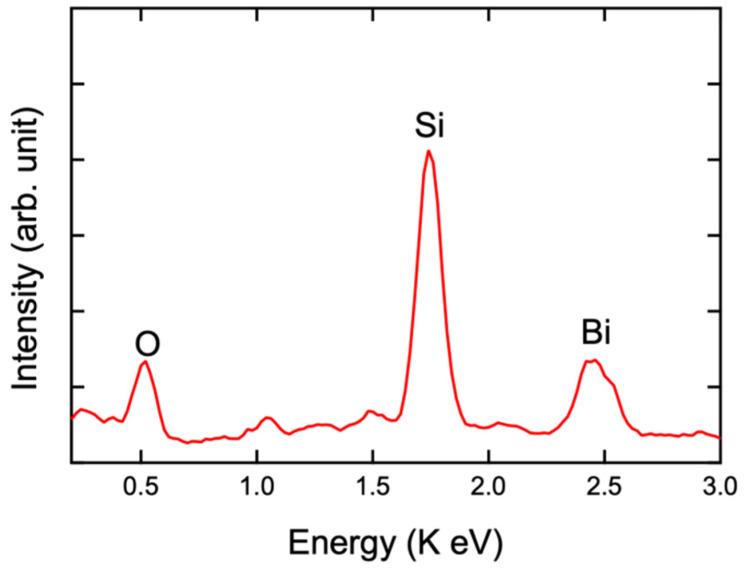
EDS spectrum of β-Bi_2_O_3_ thin film synthesized at 400 °C.

Figure 4 shows the photograph and microscopic morphology of the β-Bi_2_O_3_ film obtained at 400 °C. As seen in the figure, the β-Bi_2_O_3_ film obtained at 400 °C has a macroscopically continuous and uniform surface. The β-Bi_2_O_3_ film appears yellow. To further characterize the morphology of β-Bi_2_O_3_ films at different deposition temperatures, optical microscopy and SEM were used to observe the β-Bi_2_O_3_ films. The figure shows that the deposited β-Bi_2_O_3_ films are composed of aggregated particles, with a macroscopically continuous surface and a relatively uniform and dense film surface. The low resolution and/or inaccuracy might be caused by the amorphous impurities in the coating. In addition, we used EDS to detect the element composition of the β-Bi_2_O_3_ thin film (Figure 5). The molar ratio of Bi:O is 2:3.02, which is almost close to the theoretical value.

### 3.3. Optical Performance Analysis

**Figure 6:**
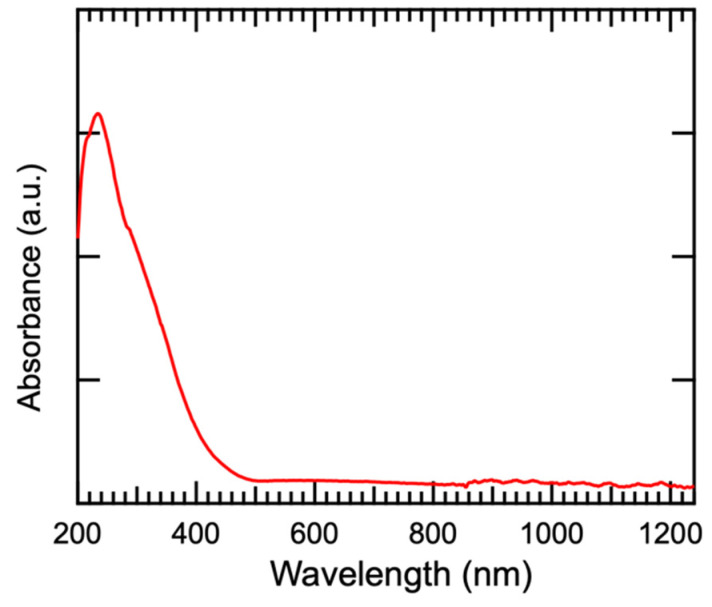
Absorption spectrum of β-Bi_2_O_3_ thin film synthesized at 400 °C.

Figure 6 shows the absorption spectrum of β-Bi_2_O_3_ thin film that β-Bi_2_O_3_ synthesized at 400 °C exhibits strong absorption of light below 400 nm wavelength, while weak absorption of high-wavelength light.

**Figure 7:**
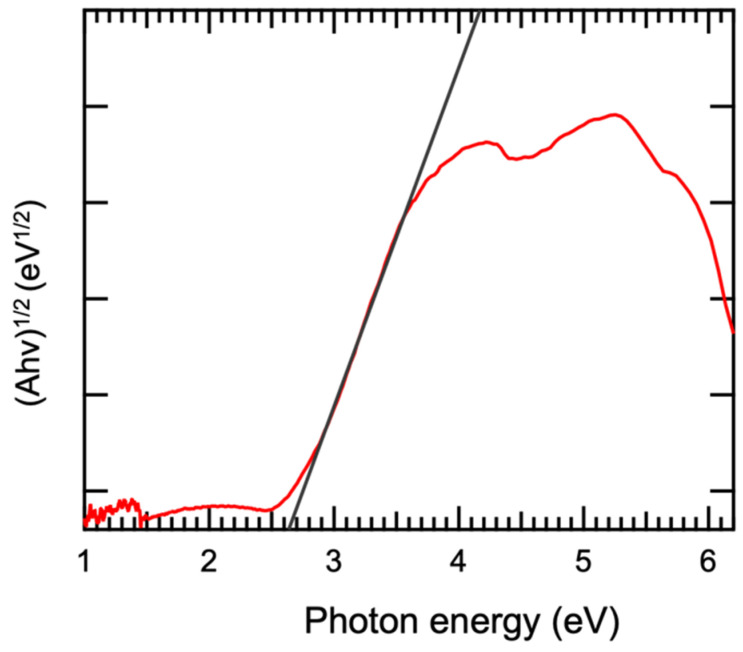
Tauc-plot of β-Bi_2_O_3_ thin film synthesized at 400 °C.

To further investigate the bandgap width of β-Bi_2_O_3_ thin films, the Tauc-plot method was used for estimation, and the calculation formula is as follows.

(1) $$\left({\alpha hv)}^{\frac{1}{n}}=B(hv-E_{g}\right)$$

where α is the absorption coefficient, Planck’s constant h = 4.13566 × 10^−15^, B is a constant, v is the incident photon frequency, E_g_ is the bandgap, and n is determined by the type of semiconductor bandgap (for a direct bandgap semiconductor n = 1/2, and for an indirect bandgap semiconductor n = 2). Based on the calculation results and previous studies, the value of n is selected. After fitting the calculated graph, the tangent at the point with the maximum slope intersects the *x*-axis at the bandgap value. As shown in Figure 7, the bandgap of β-Bi_2_O_3_ is 2.6 eV.

### 3.4. Construction and Performance Research of Photodetectors

**Figure 8:**
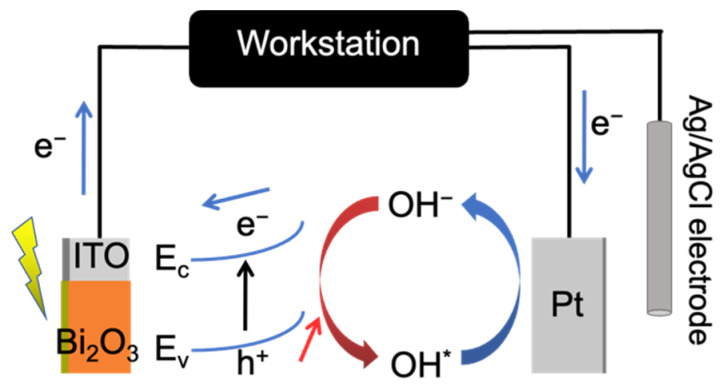
Structural diagram of β-Bi_2_O_3_ photodetector.

In the photoelectrochemical-type photodetector, the β-Bi_2_O_3_ film obtained at 400 °C is used as the working electrode, with the platinum electrode and Ag/AgCl electrode serving as the counter electrode and reference electrode, respectively. The structure of the detector is shown in Figure 8. The electrolyte affects the transport process of charge carriers and can significantly impact the performance of the constructed photoelectrochemical device. In this study, 0.5 mol/L Na_2_SO_4_ was used as the electrolyte.

The detection capabilities of the β-Bi_2_O_3_-based photoelectrochemical photodetector for light sources with different wavelengths (365 nm, 420 nm, 520 nm) and powers were characterized, as shown in Table 4.

**Figure 9:**
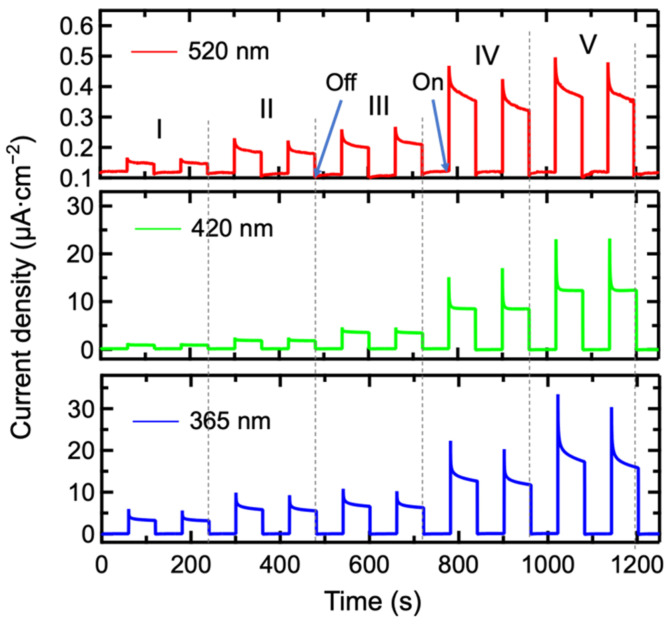
Time-resolved current graph of β-Bi_2_O_3_ photodetector illuminated by various wavelengths and power densities of illumination.

The variation of the photoresponse over time under illumination with light sources of different wavelengths and powers is shown in Figure 9. Under a bias of 0 V, the β-Bi_2_O_3_-based photoelectrochemical photodetector exhibits a significant photoelectric response to light sources ranging from 365 nm to 520 nm, indicating that the detector has self-powered characteristics and a wide detection range. At approximately low light source power density, the photocurrent value reaches around 3.3 µA∙cm^−2^ under illumination with a 365 nm light source. As the wavelength increases to 520 nm, the photocurrent value decreases to around 0.15 µA∙cm^−2^, which is related to its absorption characteristics. As the power density increases, the time-resolved curves gradually show instantaneous peaks, indicating the recombination of photogenerated charge carriers. When the recombination is more severe, the peak area is larger.

To characterize the current changes under illumination, the net photocurrent density *(I_ph_*) is introduced to measure the performance of the photodetector. The calculation formula of $I_{ph}$ is shown in Equation (2).

**Figure 10:**
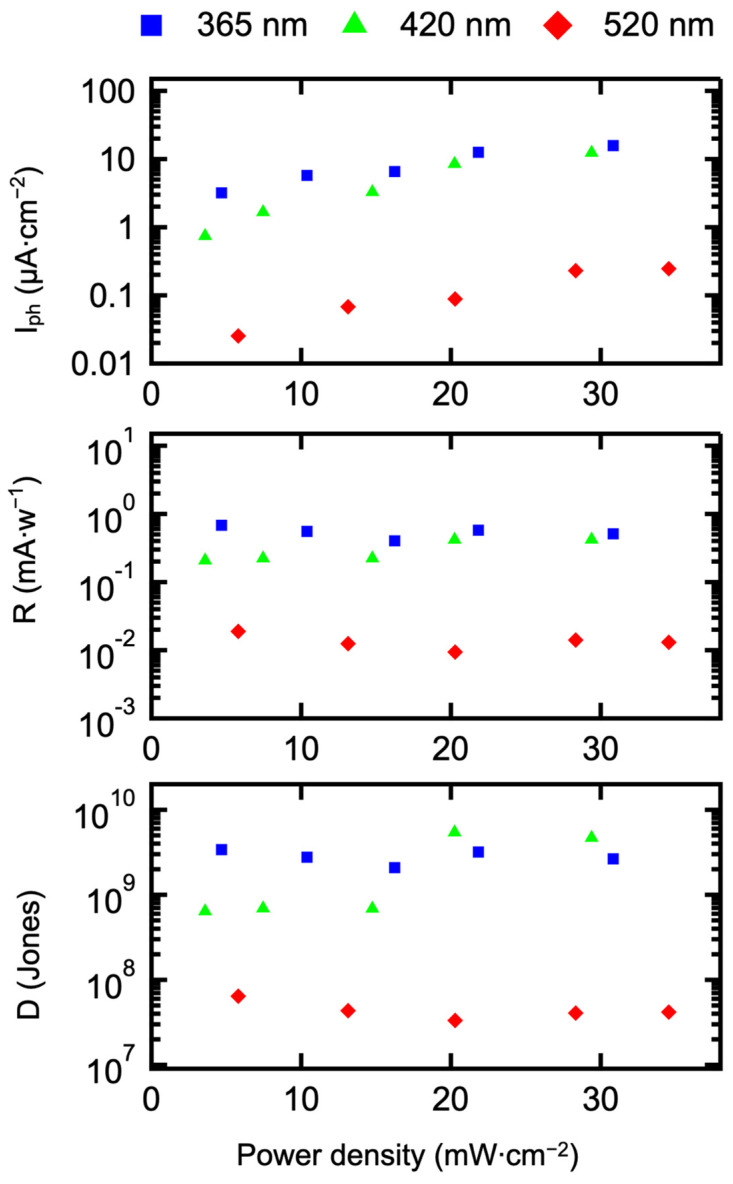
The *I_ph_*, R, and D of the β-Bi_2_O_3_ photodetector under different wavelengths and power density light irradiation.

*I_ph_* = *I_light_* − *I_dark_* (2)

where I_light_ and I_dark_ represent the photocurrent of the β-Bi_2_O_3_ photodetector under illumination and the dark current without illumination, respectively. Figure 10 shows the *I_ph_* of the β-Bi_2_O_3_ photodetector measured under different wavelengths and power densities. As shown in the figure, under illumination with a 365 nm light source, as the laser power density increases from 4.46 mW/cm^2^ to 21.64 mW/cm^2^, $I_{ph}$ increases from 3.2 μA/cm^2^ to 15.8 μA/cm^2^. This indicates that $I_{ph}$ gradually increases with increasing laser power density. This is attributed to the increased power, which increases the number of photons, causing more electrons in the valence band to transition to the conduction band, generating more photogenerated electron–hole pairs and transferring them, resulting in higher photocurrent density. Under similar laser power densities, as the light source wavelength increases from 365 nm to 520 nm, the $I_{ph}$ value decreases. This is consistent with the absorption characteristics of the β-Bi_2_O_3_ film at 400 °C, where light absorption gradually decreases with increasing wavelength.

The photodetection performance of the β-Bi_2_O_3_ photodetector at 400 °C depends on the wavelength and power of the incident light source. Responsivity (R) and detectivity (D*) can still be used to measure the performance of the photodetector, calculated as shown in Equations (3) and (4).

(3) $$R=I_{ph}/P$$

(4) $$D*=\left(R\times \sqrt{S}\right)/\sqrt{2\times q\times I_{ph}}$$

The values of R and D* obtained from calculations based on $I_{ph}$ are also shown in Figure 10, where P represents the laser power density (mW/cm^2^), S is the effective light detection area (cm^2^), and q is the electronic charge (C). The relationship between R, D*, light power, and wavelength is derived from these calculations. Under a bias voltage of 0 V and constant wavelength of 365 nm, as the power density of the light source increases, the highest responsivity R reaches 0.68 mA/W, and the highest detectivity D* reaches 3.4 × 10^9^ Jones. Under illumination conditions with wavelengths of 365 nm, 420 nm, and 520 nm, the responsivity R values obtained are 0.68, 0.21, and 0.02 mA/W, respectively. The detectivity D* values obtained under illumination conditions with wavelengths of 365 nm, 420 nm, and 520 nm are 3.4 × 10^9^, 6.42 × 10^8^, and 6.45 × 10^7^ Jones, respectively. This indicates that under constant wavelength conditions, the detection capability of the β-Bi_2_O_3_ photodetector for photoelectrons decreases inversely with light power density. In other words, as the light power density increases, the detection capability of the β-Bi_2_O_3_ photodetector weakens. This phenomenon occurs because under high power density irradiation, the number of charge carriers in the β-Bi_2_O_3_ structure increases rapidly, leading to increased scattering between carriers, hindering their transfer and transport. This also increases the probability of electron–hole recombination, significantly increasing the recombination rate of carrier pairs per unit time. Furthermore, as the wavelength of light increases, photon energy decreases gradually, reducing the absorption capacity of the β-Bi_2_O_3_ film for light. Therefore, both the responsivity and detectivity of the β-Bi_2_O_3_ photodetector decrease accordingly.

**Figure 11:**
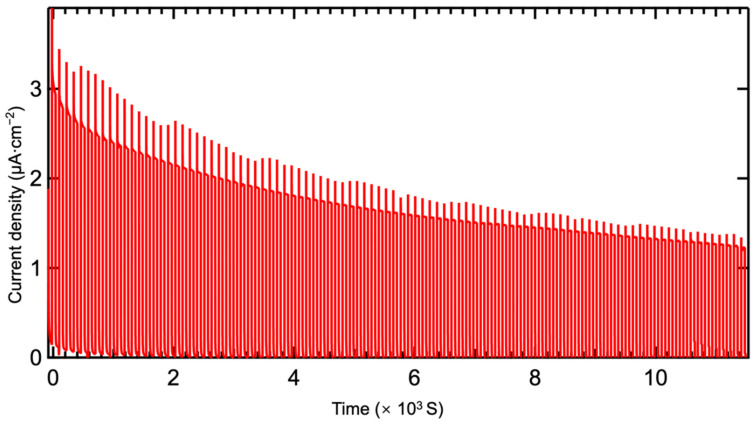
Long-term stability for β-Bi_2_O_3_ synthesized at 400 °C with PEC-type photodetector.

**Figure 12:**
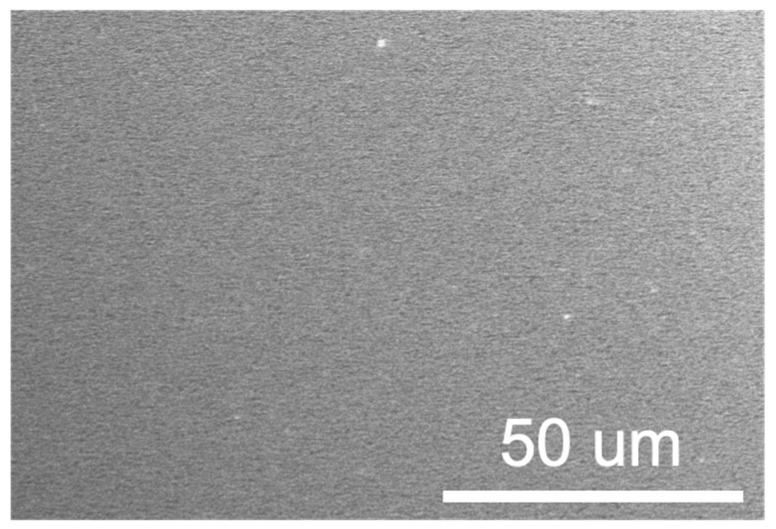
SEM image of β-Bi_2_O_3_ thin film after the long-term stability test.

In practical applications, long-term stability is a critical metric for evaluating the performance of photoelectrochemical photodetectors. To investigate the stability of the β-Bi_2_O_3_-based photoelectrochemical photodetector at 400 °C, this study utilized a 365 nm LED light source with a laser power density of 4.5 mW/cm^2^ and a cycling interval of 10 s for long-term i-t stability testing, as shown in Figure 11. After the first cycle, the photocurrent density was 2.8 μA/cm^2^. Following 500 on–off cycles, the photocurrent density decreased to 1.3 μA/cm^2^, indicating a decrease of approximately 1.5 μA/cm^2^. This demonstrates that after one hour of operation, the photodetector maintained approximately 45% of its initial photocurrent density, indicating acceptable on–off stability. After the PEC stability, we checked the surface morphology of β-Bi_2_O_3_ thin films that there were no clear changes observed (Figure 12).

**Figure 13:**
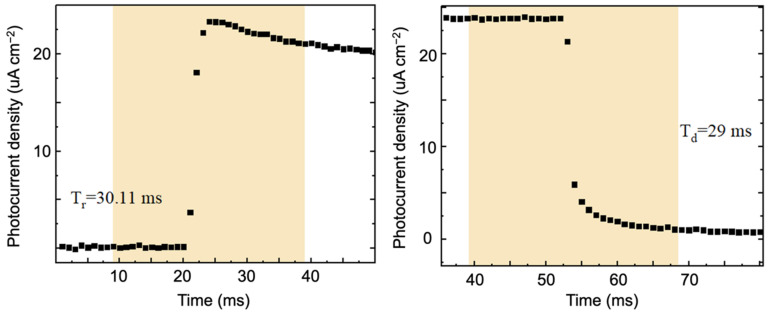
Response time graph of β-Bi_2_O_3_ photodetector.

Response time is primarily used to measure the speed at which a detector responds to incident light. It refers to the time it takes for the detector’s response output to rise to stability or fall back to the pre-irradiation value in response to a rectangular light pulse. A shorter response time indicates a faster detection speed for the detector. To investigate the response speed of the PEC photodetector based on the β-Bi_2_O_3_ to light, this study tested the response time at a wavelength of 365 nm and a power density of approximately 30 mW/cm^2^, with a testing step of 1 ms. The results, shown in Figure 13, depict the response time of the β-Bi_2_O_3_ photodetector. From the graph, it can be observed that the response speeds are τ_rise_ = 30.11 ms and τ_decay_ = 29 ms, demonstrating an ultra-fast response speed compared to other materials, promising for achieving ultra-fast detection. Compared to bismuth-based semiconductor PEC-type photodetectors, the β-Bi_2_O_3_ PEC-type photodetector exhibited comparable and even superior photodetection performance in terms of responsivity, response time, and stability (Table 5).

## 4. Conclusions

In this paper, we synthesized the β-Bi_2_O_3_ thin films by the mist chemical vapor deposition method. The β-Bi_2_O_3_ film deposited at a temperature of 400 °C has high purity, a uniform and continuous surface, and a bandgap width of 1.35 eV. β-Bi_2_O_3_-based photoelectrochemical photodetectors have a wide light absorption range, self-driving properties, and high stability. The highest responsivity R and detection rate D* reached 2.84 mA/W and 6.01 × 10^10^ Jones, respectively. The response speeds of PEC photodetectors based on β-Bi_2_O_3_ at a wavelength of 365 nm and a power density of approximately 30 mW/cm^2^ are τ_rise_ = 30.11 ms and τ_decay_ = 29 ms, respectively, showing an ultrafast response speed and promise for ultrafast detection. Additionally, carbon residue might occur during the deposition of Bi_2_O_3_ so post-annealing might be beneficial for the improvement in film quality. In addition, optimization of electrode architecture would be another strategy for the enhancement of PEC-type photodetection performance. Thus, this study would promote the practical applications of bismuth-based semiconductors.

## Figures and Tables

**Table 1:** The summary of the preparation methods for β-Bi_2_O_3_ thin films.

Method	Ref.	Temperature	Precursor	Time
Solid-state reaction	[14]	450–600 °C	Bi_2_SiO_5_	2 h
Solution methods	[15]	300–500 °C	Bi_38_O_45_(OMc)_24_(DMSO)_9_·2DMSO·7H_2_O	75 min
CVD	[16]	500–800 °C	Bismuth carbamate	10–40 min
PVD	[12]	300–600 °C	Bi (001) film, BiO_x_, amorphous film	30–35 min

**Table 2:** Experimental materials and manufacturers.

Chemical Reagents	Chemical Formula	Purity	Manufacturer
25 wt% bismuth(III) 2-ethylhexanoate (C_24_H_45_BiO_6_)		≥99.5%	Aladdin (Shanghai, China)
N. N-Dimethylformamide	C_3_H_7_NO	≥99.50%	National Pharmaceutical Group (Beijing, China)
High purity nitrogen gas	N_2_	≥99.90%	Wuhan Xiangyun Company (Wuhan, China)
Anhydrous ethanol	C_2_H_5_OH	≥99.00%	National Pharmaceutical Group (Beijing, China)
Deionized water	H_2_O	≥99.99%	Self-prepared
Acetone	C_3_H_6_O	≥99.00%	National Pharmaceutical Group (Beijing, China)

**Table 3:** Crystal structure parameters calculated from the XRD pattern.

Parameter	Value
a = b	0.770 nm
c	0.568 nm
Crystallite size	18.90 nm

**Table 4:** Laser power density at different wavelength power percentages (mW/cm^2^).

Wavelengths	I	II	III	IV	V
365 nm	4.46	6.55	10.12	15.32	21.64
420 nm	3.58	7.45	14.75	20.34	29.40
520 nm	5.79	10.76	13.13	20.28	28.33

**Table 5:** Photodetection performance of recently reported PEC-type photodetectors.

Materials	Ref.	Electrolyte	Light (nm)	Voltage (V)	R (mA/W)	τ_r_/τ_d_ (ms)	Stability (per Cycle Decrease)
Bi_2_O_2_S	[27]	0.5 M KOH	Sun	0.6	0.23	80/70	–
BiSeI	[28]	0.2 M Na_2_SO_4_	600	0.5	2.88	7/16	0.09%
Bi_2_O_2_Se	[29]	Na_2_SO_3_	365	0	14.24	9/12	–
Bi_4_O_5_I_2_	[30]	0.5 M Na_2_SO_3_	420	0	19.4	1.9/3.0	0.12%
Bi_2_O_2_S/GO	[31]	KOH/PVA	Sun	0	0.035	420/2300	0.05%
OV-Bi_4_O_5_I_2_	[32]	0.5 M H_2_SO_4_	365	0	18.2	1.4/1.0	0.082%
Bi_13_S_18_I_2_	[33]	0.5 M Na_2_SO_4_ + 0.7M Na_2_SO_3_	0	20.2	2.0/1.0	0.05%	–
β-Bi_2_O_3_	This work	0.5 M KOH	365	0	2.84	30.1/29.1	0.09%

## Data Availability

All data generated or analyzed during the study are available from the corresponding author upon request. The data are not publicly available due to privacy.
